# Impact of the Optimistic Perspective on the Intention to Create Social Enterprises: A Comparative Study Between Portugal and Spain

**DOI:** 10.3389/fpsyg.2021.680751

**Published:** 2021-05-12

**Authors:** Clara Margaça, Brizeida Raquel Hernández-Sánchez, Giuseppina Maria Cardella, José Carlos Sánchez-García

**Affiliations:** Department of Social Psychology and Anthropology, Faculty of Psychology, University of Salamanca, Salamanca, Spain

**Keywords:** optimism, spirituality, Spain, Portugal, intention, social entrepreneurship

## Abstract

Social entrepreneurship (SE) enables business consolidation, combined with the production of positive impact and improvements in society. Aligned with 2030 Agenda for the achievement of the United Nations Sustainable Development Goals, it is important to clarify the role of social entrepreneurs, as they are making visible the impact of their creative ideas in several areas, from civic engagement to the environment, health and learning. The main purpose of this study is to specify a model of social entrepreneurial intention (SEI) and explore it by country, based on the dimensions of the Theory of Planned Behavior and how these are mediated by spirituality and optimism. With a convenience sample of 1476 Portuguese and Spanish students, Structural Equation Modeling (SEM) was used. For a deeply understanding, variables within the model were compared by country using *t*-rest, and multivariate analysis was done by each one separately. The mean comparison between countries, demonstrated that there are differences only between perceived behavioral control (PBC), Spirituality, Optimism and SEI, with Portuguese students scoring the highest values, in all variables except Optimism. A mediation path was carried out, and Spirituality mediate a significant effect between the three TPB dimensions in Portuguese students, but not in students from Spain. Finally, after discussing the results, theoretical and practical contributions are analyzed, with regard to the field of SE in Portugal and Spain, and alternatives are pointed out for a more social and sustainable entrepreneurial future.

## Introduction

The phenomenon of social entrepreneurship (SE) emerges in the context of crisis and social, economic, and environmental challenges that contemporary societies have been facing. The relationship between SE and the generation of social value has as a central figure the individuals/actors, and their actions are responsible for reconstructing and transforming society (Sastre-Castillo et al., 2015; Muñoz and Kibler, 2016). The great difference of social entrepreneurs lies in the reach of the social impact they manage to generate, as well as in the multiplicity of approaches that are applied to solve social problems (Nicholls, 2006). Over the years, the literature has provided insights into the complex process underlying entrepreneurial activity, through studies on cognitive factors (e.g., motives to undertake) and intention (Krueger et al., 2000; Autio et al., 2001; Peterman and Kennedy, 2003). This cognitive perspective is invaluable because, as important as studying the economic impact of entrepreneurship on societies, is to understand, at the individual level, the formation of new ventures and their structures (Grégoire et al., 2011; Sánchez et al., 2011). According to Bornstein (2004) and Bornstein and Davis (2010), SE started out as an activity promoted by individuals who combined their ability to idealize a better society with their ability to act, transforming ideas into concrete initiatives. Thus, it is also important to deepen the understanding of the factors that guide and influence social entrepreneurial intentions (SEI).

Currently, according to data from the Global Entrepreneurship Monitor (GEM), SE involves between 2.5 and 5% of the European population, and is an important driver for increasing inclusion, justice, and prosperity (GEM, 2020). In Portugal, for example, SE lives with the scenarios of economic crisis and, consequently, of high unemployment rates (Casaqui, 2014). In this country, the social sector represents 3% of the Gross Value Added, however, there is still a lack of a legal framework for the concept of social enterprise, which allows them to maximize their eligibility for certain financing opportunities (Nova SBE, 2020). The latest GEM Special Report on Social Entrepreneurship reported that, in Spain, the number of companies with social objectives has increased (0.9%), but the indices continue well below the Portuguese (2.7%) and the European (2.98%) (Bosma et al., 2016).

Previous research reports that entrepreneurial intention is the result of individual and contextual factors (Giacomin et al., 2016). In other words, culture can shape individual dispositions and, therefore, the intention to have a business of their own (Thornton et al., 2011; Shinnar et al., 2012). In a society where it is possible to enjoy respect, there is an environment more favorable to the entrepreneur potential (Giacomin et al., 2016). Swail et al. (2014) acknowledge that “the degree to which individuals feel motivated to seek entrepreneurial opportunities will be reflected in the belief that entrepreneurship is socially acceptable and that entrepreneurs themselves are respected members of the community” (p. 864). That is, the differences in entrepreneurial intention are explained by the valuation of entrepreneurship in society (Liñán et al., 2011). All societies have a unique set of values, which was built through its history and must be understood, that is, each society has its own culture. Thus, it is possible that factors that influence the social entrepreneur’s intentions may vary from culture to culture or from region to region. A study by Liñán and Chen (2009) highlighted that the beliefs, attitudes and perceived behavioral control (PBC) associated with entrepreneurship vary between Spain and Taiwan, for instance. When comparing two regions in Spain, Liñán et al. (2011) revealed that entrepreneurs are more valued in the most economically developed region. The perception of barriers to entrepreneurship was also different in three different countries (Shinnar et al., 2012). Liñán et al. (2013), they also pointed out differences in the entrepreneurial intention between Spain and Great Britain. Several studies (e.g., Monllor, 2010; Patzelt and Shepherd, 2011; Lough and McBride, 2013) analyzed the process of creating a social enterprise, focusing on components such as the recognition of opportunities and motivations, but few evaluated the intentions (e.g., Krueger et al., 2007; Nga and Shamuganathan, 2010). In addition, the latter were confined to the North American or Chinese context (Yang et al., 2015). The purpose of this article is to identify more deeply the factors that influence SE and, in a specific way, to examine how these factors may differ in the cultural contexts of Portugal and Spain.

The need to study the influence of different cultures on SEI becomes clear. One of the objectives of this article is to fill this gap by using a sample from the Iberian Peninsula. The geographical, historical or even linguistic proximity does not make the Iberian Peninsula a culturally homogeneous territory; however, we can find some similarities in several aspects. After being severely punished by the global crisis of 2008, unemployment in Portugal was 16.8%, the public deficit exceeded 3% and the debt reached 132% of GDP in 2013. After the years of the presence of the troika and the austerity of the IMF, the country returned to breathe and reap the rewards of previous investments. Investment rose 9%, largely with resources from private and foreign sources, and unemployment fell to 8.9% (PORDATA, 2014). As the same way as Portugal, Spain also approves an Austerity Plan for 2011–2013, after the 2008 housing bubble. In 2010, according to National Institute of Statistic (INE) from Spain, the economic downturn is confirmed, with the country losing 3.6% of its wealth and the rate of unemployment reaches 20% (Instituto Nacional de Estadística [INE], 2013). Several authors acknowledge that the creation of new businesses could be particularly important during periods of economic recession because, when successful, it helps generating new jobs, spreading innovation and providing support to economy (Dana and Wright, 2004; Ahmed et al., 2010).

The economic crisis and the resulting austerity policies have had a profound impact on the structure and patterns of the labor market. In Portugal, in 2011, for example, the Strategic Program + E + I (More Entrepreneurship, More Innovation) was created and, in 5 months, registered more than 4000 applications. This adhesion mirrors the Portuguese people’s growing desire to undertake; however, for experts it was also the direct result of a country in crisis, where employment opportunities have suffered a significant drop. In Spain, entrepreneurial activity continues to increase (e.g., from 5.2% in 2016 to 6.4% in 2019) and remains on the path to recovery toward pre-crisis figures (7.6% in 2007).

The literature says that crisis scenarios lead to entrepreneurial behaviors (Bullough et al., 2014; Devece et al., 2016). The current moment, triggered by the pandemic, has created (and will continue to create) many social challenges for which we are not prepared; thus, it will be necessary to develop innovative solutions. Despite having different characteristics, the presence of COVID-19, in these two countries, has proven that the worsening of social problems opens the door to social entrepreneurs and to the creation of powerful and innovative solutions, particularly in affected areas. In Portugal, for instance, to fill social isolation, the SOS Vizinho project was quickly set up, which helps to signal and support groups at risk in the neighborhood with the purpose of distributing essential goods, consequently they do not need to leave their homes frequently. The social response to the coronavirus crisis was shown in Spain, for example, through Farmidable, which allows access to local products, giving the purchase free of charge to the most vulnerable people, mainly the elderly and high-risk groups.

Currently, the study on entrepreneurship has revealed another type of interest; not only the study of the economic aspect, but also that this is not the main reason for starting an entrepreneurial activity (Katz, 1992; Amit et al., 2001). These new outputs develop a new and greater understanding of the motivations of entrepreneurs, as well as their personal values and belief structures, which influence their entrepreneurial decision (Bird and Schjoedt, 2009; Krueger, 2009; Smith et al., 2019). The entrepreneurship process begins long before any economic gain. When studying entrepreneurship, it is also important to consider the idiosyncrasies of the main actor in the process – the individual’s internal and personal values, motivational needs, beliefs and desires. The individual’s spiritual orientation positively influences the decision-making process of becoming an entrepreneur, through resilient coping mechanisms. These people who attach importance to spirituality have better levels of health, productivity, happiness and a better ability to deal with stress and adversity (Balog et al., 2014; Riaz et al., 2017). Spirituality comes to be seen as an emerging theme in the field of social sciences and administration. For instance, several authors (e.g., Balog et al., 2014; Nandram, 2016; Smith et al., 2019) recognized that it is plausible to study the influence of spirituality on management when it is defined in terms of attitudes, behaviors and practices.

## Theoretical Background and Conceptual Model

### Social Cause as Intention

Entrepreneurial intention is recognized as an individual’s conscious conviction to direct attention toward a certain goal and achieve it in the future (Bird, 1988; Thompson, 2009) and also as the result of conscious thinking and complex cognitive processes (Krueger et al., 2000). It is consensual for several authors (e.g., Kolvereid and Isaksen, 2006; Krueger and Brazeal, 2017) that the entrepreneurial intention is perceived as a prerequisite to carry out a general entrepreneurial activity and for SE in particular (Mair and Noboa, 2006). Similarly, the SEI can be understood as mental orientation, such as a belief, desire, hope and determination of a person to set up a new social enterprise (Tran and Von Korflesch, 2016). There are two dominant models that explain the SEI: Model of Social Intention Formation (Mair and Noboa, 2006) and the Theory of Planned Behavior (TPB) (Ajzen, 1991). Based on the Entrepreneurial Event Model by Shapero and Sokol (1982), the Model of Social Intention Formation was developed specifically to predict SEIs (Mair and Noboa, 2006). This model suggests that the intention to create a social activity is influenced by a person’s perceived ability to start a social enterprise and their perceived convenience. On the one hand, the perceived viability consists in the belief that the individual has all the necessary cognitive and motivational capacities (self-efficacy) and social capital to create a social enterprise (Wood and Bandura, 1989). On the other hand, perceived convenience is made up of the ability to detect and understand other people’s emotional and affective states and to react accordingly – empathy (Mehrabian and Epstein, 1972) and the motivation to help other people achieve a common goal – moral judgment (Mair and Noboa, 2006). The Theory of Planned Behavior (TPB) has been applied, exhaustively, as a framework for many studies that seek to explain the complex cognitive processes that lead to the creation of the company (Krueger et al., 2000; Liñán, 2008) and, consequently, of entrepreneurial intention and behavior (Maes et al., 2014). Several studies (e.g., Mair and Noboa, 2005, 2006) have proven their application to the prediction of entrepreneurial intention, and others studies have also demonstrated that the TPB model is applicable as a theoretical framework for SEI (Ernst, 2011; Forster and Grichnik, 2013; Hockerts, 2017; Tiwari et al., 2017). A complete review of the literature on factors that affect the social entrepreneur’s intentions elaborated by Ahuja et al. (2019) reveals that many of the factors studied could be grouped under a broad construct of personality, for example, empathy (Mair and Noboa, 2006), proactive personality (Prieto, 2010), or ability to take risks (Irengün and Arikboga, 2015).

The TPB (Ajzen, 1991) suggests that the formation of an intention is influenced by three different constructs: (i) attitude toward behavior (AT) – based on behavioral beliefs of a person who evaluate whether the consequences of an action are judged negatively or positive; (ii) subjective norms (SN) – measured the social pressure of other important people in a person’s life can influence personal decisions and; (iii) PBC – on the one hand, the perceived ease of performing this action (self-efficacy) and, on the other hand, the necessary resources and control over the execution of these actions (controllability).

The main motivation of the social entrepreneur is to find the right opportunity to help and solve problems in society (Mair and Noboa, 2006), driven by their belief system and values and their skills. An individual with a SEI has “self-acknowledged conviction by a person that they intend to become a social entrepreneur and consciously plan to do so at some point in the future” (Thompson, 2009, p. 676). The societies have their own characteristics and, therefore, a person’s individual history and exposure to certain social problems, their attitudes and perceptions about it (Mair and Noboa, 2006) can have an influence on their values. This can make them more receptive and aware the others’ issues, which may trigger their interest in creating a social activity (Monllor, 2010; Patzelt and Shepherd, 2011; Miller et al., 2012) and produce social change. Regarding to SEI, Kruse et al. (2020) acknowledge that TPB suggests that people who judge a career as a social entrepreneur positively (attitude), have social support from other important people (subjective norms) and the belief that they are capable of creating a social enterprise (PBC) have a high intention of creating a social entrepreneurial activity. Therefore, the first set of hypotheses is:

H1: For both countries– PBC has a significant and positive effect on SEI, which is not significantly different from each other.
H2: For both countries – AT has a significant and positive effect on SEI, which is not significantly different from each other.
H3: For both countries – SN has a significant and positive effect on SEI, which is not significantly different from each other.

As far as SE is concerned, Portugal presents important examples, such as Color Add in the inclusion of people with color blindness or Speak in the integration of refugees and immigrants, among many others. These innovative ideas inspired a new public policy and made the country as an international reference in supporting social innovation. For its part, Spain has a very similar path relatively to the concern in suppressing social needs. Change Dyslexia is a platform that democratizes access to detection and support of dyslexia to overcome the barrier of economic resources. Or the Reticare that produces the world’s only eye shield designed and patented to absorb high-energy light from digital devices, once this is the world’s leading cause of blindness. In the Iberian Peninsula, this sector is gaining a new role, particularly in the current economic situation, presenting entrepreneurial and innovative solutions to respond to the pressing needs of the populations, namely of an economic, social and environmental nature. Thus, although the two countries have cultural heterogeneity, both meet requirements for comparative research.

## Being Optimistic: True or False Expectations?

Social entrepreneurship (SE) is broadly understood as a practice that aims to create and sustain social change, an innovative, social value creating activity that can occur within or across the non-profit, business, and public sectors (Austin et al., 2006). SE is a phenomenon of complex approach, since it refers to a specific work and social orientation, focused on the development of labor and social projects that cannot be classified only as traditional entrepreneurship. It involves developing, executing and sustaining initiatives aimed at overcoming a social difficulty, and the achievement of a common benefit for a human group, either through business or social-community activities (Pomerantz, 2003). The main difference proposed between entrepreneurship and SE is about the preponderance of social objectives over economic ones (Cohen et al., 2008), as well as about the most diverse sustainability mechanisms in the case of SE (Tracey and Phillips, 2007).

Several researchers have acknowledged that optimism can play a significant role in entrepreneurship (Trevelyan, 2008; Storey, 2011; Madari et al., 2019) and is considered as a requirement for someone to become an entrepreneur (Dushnitsky, 2010). However, the relationship between optimism and SEIs has been neglected. Thus, another objective of this study is to evaluate whether optimism has the same applicability in SEIs in these two cultural contexts. According to Collins (2007), optimism involves positive expectations and results, it is associated with the ability to make positive cognitive assessments and, then, to make active and engaged coping efforts to deal with stress, highlighting positive aspects of what happened. Peterson (2000) acknowledge that, in general, the research highlights that optimism is connected to positive results. In addition, optimism is also associated with a positive mood, as well as with high levels of confidence in an individual’s projections (Scheier and Carver, 1992). Intentions reflect the motivational factors that influence behavior (Krueger, 2009), and individuals with high levels of optimism and the belief that they are able to influence results indicate how much they are willing to try to perform the behavior (Urban, 2010).

Some authors have found optimism to be a driver for venture creation, as it taps into the perception that their projects will have success (e.g., Ozaralli and Rivenburgh, 2016); however, a study with Spanish students (Ward et al., 2019) revealed that optimism was not significant for intentions, despite the positive effect. Optimism is regarded as a personality trait and is linked to positive outcomes in stress and coping (Chapman and Chi, 2017). Peterson (2000) demonstrated that optimism increase persistence, commitment and creativity (Li and Wu, 2011). Given its characteristics, optimism is beneficial and decisive to decision-making to become a social entrepreneur; in order that, an optimistic entrepreneur is more likely to successfully carry out an activity and persist in the face of obstacles (Trevelyan, 2008). This leads us to the second set of hypotheses:

H4: Optimism mediate the positive effect of PBC on SEI, which is stronger in students from Portugal.
H5: For both countries – Optimism mediate the positive effect of AT on SEI, which is not significantly different from each other.
H6: Optimism mediates the positive effect of SN on SEI, and the effect is significantly stronger in students from Portugal.

## The Spiritual Side

Entrepreneurship is an experience guided by personal values (Morris and Schindehutte, 2005; Zsolnai, 2014), through which the individuals places a deep level of personal meaning, driven mainly by their internal values (Kinjerski and Skrypnek, 2004), such as spirituality. According to Strack and Fottler (2002, p. 6), “spirituality is a fundamental dimension of human existence, being as real as any other concept.” It is independent of any religion or belief system, considered as a complex, multi-cultural and multi-dimensional concept (Zsolnai and Illes, 2017), and possess a social basis and a social dimension (Oman, 2015). Spirituality is considered as a set of capabilities and abilities that make individuals capable of solving problems and reaching goals in life (Rust and Gabriels, 2011), and it is a search for the sacred (Zinnbauer and Pargament, 2005). It refers to an inner experience of an individual who discovered the meaning and purpose in life, and can also be understood as the capacity to find and construct meaning about life and existence and to move toward personal growth, responsibility, and relationship with others (Myers and Williard, 2003). Spirituality came to be seen as an emerging theme in the field of social sciences and administration. Several authors (e.g., Balog et al., 2014; Nandram, 2016; Smith et al., 2019) recognized that it is crucial to study its influence on management when it is defined in terms of attitudes, behaviors and practices. Understanding entrepreneurship in the light of spirituality has been gaining increasingly interest in the academic field (Candland, 2000; Fernando, 2007; Dana, 2010; Smith et al., 2019). Studying the influence of spirituality allows a new understanding of how an individual’s personal values and beliefs can impact the decision to become an entrepreneur, as well as the crucial characteristics of the entrepreneurial process, such as the recognition of opportunities and the ability to resist obstacles (Smith et al., 2019). The entrepreneurship process begins long before any economic gain. When studying entrepreneurship, it is also important to consider the idiosyncrasies of the main actor in the process – the individual’s internal and personal values, motivational needs, beliefs and desires. The individual’s spiritual orientation positively influences the decision-making process of becoming an entrepreneur, through resilient coping mechanisms. These individuals who attach importance to spirituality have better levels of health, productivity, happiness and a better ability to deal with stress and adversity (Balog et al., 2014; Riaz et al., 2017).

Entrepreneurs have the opportunity to contribute to a better functioning of society, integrating their personal values to work (Kauanui et al., 2010). The same authors distinguish entrepreneurs in two types: (1) “make me whole”: they have a passion for their work and express concern for others and the environment, moved spiritually; and (2) “money is king”: obsession with efficiency, production and accumulation of capital (and the resulting idolatry of money). Based on their empirical findings, Kauanui et al. (2010) conclude that spiritually oriented entrepreneurs, in comparison to financially oriented ones, benefit from a sense of joy, which provides important insights into the importance of spirituality.

By examining the connection between spirituality and entrepreneurship, we are on the path to understanding, in more depth, the role of personal values and beliefs and their influence in the process of creating an entrepreneurial activity (Balog et al., 2014). Waddock and Steckler (2012) concluded that a set of social entrepreneurs was guided by a diverse sense of hope and purpose to make a difference in society. These characteristics are covered by the concept of spirituality. In order to explain and provide support for the spiritual side of human motivation, another objective of this article is to explain if spirituality can act as a trigger in the decision-making to become a social entrepreneur.

H7: For both countries – Spirituality has a significant positive effect on SEI, which is not significantly different from each other.
H8: Spirituality mediates the positive effect between PBC and SEI, which effect is significantly stronger in Portuguese students.
H9: For both countries – Spirituality mediates the positive effect between AT and SEI, and is not significantly different from each other.
H10: For both countries – Spirituality mediates the positive effect between SN and SEI, and is not significantly from each other.

**FIGURE 1:**
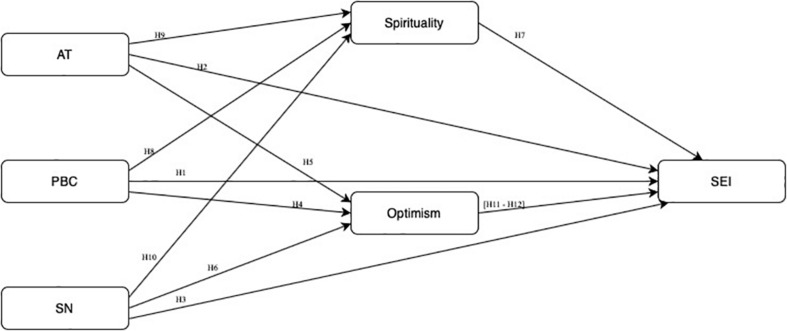
Structural model.

For both countries – PBC (H11) and AT (H12) positively increase Optimism, and one of the reasons is this makes individuals more motivated to deal with the undertake process, having a positive effect on their SEI. Figure 1 represents the proposed structural model.

## Materials and Methods

### Sample and Procedure

#### Sampling

Study participants were selected by stratified sampling, because, despite their geographical, proximity Portugal and Spain have profound political, social and cultural variations, and also because a specific area of study (namely the business area) was not specifically focused. Hence, one of the first objectives was to achieve the largest possible geographical cover of the two countries and academic areas. Data collection was carried out from December 2018 to February 2019, through the collaboration of public relations or communication offices of the various universities in both countries. At first, the students agreed to an informed consent, with the guaranteed of the protection of their data, which includes anonymity and confidentiality. The students received the questionnaire by e-mail and responded using an online platform. Before the questions, information was given about the purpose of the study and how they should respond. Although there is no time limit for completing the questionnaire, the estimated time for completing it was 15 min.

#### Participants

The resulting sample comprised 1476 university students from both countries. For the Portuguese sample, data was collected from students at twenty-one universities and seven polytechnic institutes in the continent and islands. It comprised 644 respondents engaged in courses in different areas (31.2% health-related students, 18.3% business/management-related students, and 14.6% law and social-related students). Of these respondents, 68.9% were female and 31.1% were male, with an average age of 25 years.

The Spanish sample comes from 34 universities, also from the continent and the islands, with a total of 832 participants. Spanish students were mostly engaged in law and social sciences-related area (27.8%), health-related area (26.3%), and technologies-related area (10.2%). Of these 832 students, 72.2% were female and 27.8% were male, with an average age of 23 years old. Table 1 presents the sociodemographic results of the two countries.

**TABLE 1:** Sociodemographic characterization.

		**Portugal**		**Spain**		**Total**	
		***N***	**%**	***N***	**%**	***N***	**%**
Gender	Female	444	68.9	608	73.1	1476	100
	Male	200	31.1	224	26.9		
Work experience		310	48.1	421	50.6	731	49.5
Indep. work experience		187	29.0	54	6.5	241	16.3
Health		201	31.2	219	26.3	420	28.5
Technologies		60	9.3	85	10.2	145	9.8
Agriculture and natural resources		33	5.1	27	3.2	60	4.1
Architecture, plastic arts, and design		31	4.8	61	7.3	92	6.2
Education		41	6.4	65	7.8	106	7.2
Law and social sciences		94	14.6	231	27.8	325	22.0
Business/Mang		118	18.3	71	8.5	189	12.8
Humanities		45	7.0	51	6.1	96	6.5
Sports and performative arts		21	3.3	22	2.6	43	2.9

#### Instruments

This research uses the Entrepreneurial Orientation Questionnaire (Sánchez-García, 2010), which presents statements that must be answered in range metrics; that is, a Likert scale from 1 to 5. The scale has the specific objective of measuring entrepreneurial skills and related attitudes: PBC (6 items), Attitude toward Entrepreneurship (10 items), Subjective Norm (4 items), Social Entrepreneurship Intention (25 items), and Optimism (10 items).

Perceived behavioral control (PBC α = 0.77) is defined as the perception of the ease or difficulty of becoming an entrepreneur; the feeling of confidence and ability to control and carry out a behavior to create a company. There are some examples of the items: starting a business would be easy for me; or I know how to develop an entrepreneurial process. Both are examples of self-efficacy and controllability, respectively. Attitude toward entrepreneurship (AT α = 0.93) is deeply connected to intentional and volitional behavior, beliefs, attitudes (Elfving, 2008) and a set of skills. ATE refers to “the degree to which a person has a favorable or unfavorable appraisal of the behavior under scrutiny” (Fini et al., 2012, p. 390). As an example, we highlight one of the items; I feel very competent and confident that I could identify market opportunities for a new business. Subjective Norm (SN α = 0.75) refers to the perceived social pressure to perform or not a behavior and the perception of what the important people in the life of an individual might think about the decision to become an entrepreneur. This variable is commonly measured by asking participants to what extent they think their closest ones would support them in engaging in entrepreneurial activities (Ajzen, 2002; Liñán and Chen, 2009).

The Social Entrepreneurial Intention (SEI α = 0.77) reveals that an individual consciously plans to create a social enterprise at some point in the future. An item example is: “When I think of socially disadvantaged people, I try to put myself in their place.”

Optimism (Opt α = 0.71) frames the level of agreement in which a person believes that their future holds positive outcomes, or that there is a positive side of every experience. An item example is: “No matter how bad things can go, I always find something positive.”

And to operationalized Spirituality (Spirit α = 0,98) we used modified six-item Intrinsic Spirituality Scale (Hodge, 2003) that measure the degree to which spirituality functions as an individual’s master motive, for theistic and non-theistic populations, both within and outside of religious frameworks. The scale uses a sentence completion format to measure various attributes associated with spirituality, that is, an incomplete sentence fragment is provided, followed directly below by two phrases that are linked to a scale ranging from 0 to 10. The range provides with a continuum on which to reply, with 0 corresponding to absence or zero amount of the attribute, while 10 corresponds to the maximum amount of the attribute (e.g., In terms of the questions I have about life, my spirituality answers; 0 – no questions and 10 – absolutely all my questions). This instrument was translated and adapted for the Portuguese and Spanish languages. This scale measures the degree to which spirituality functions as an individual’s master motive, for theistic and non-theistic populations, both within and outside of religious frameworks. It uses a sentence completion format to measure various attributes associated with spirituality; that is, an incomplete sentence fragment is provided, followed directly below by two phrases that are linked to a scale ranging from 0 to 10. The range provides with a continuum on which to reply, with 0 corresponding to absence or zero amount of the attribute, while 10 corresponds to the maximum amount of the attribute (Hodge, 2003).

In this research were used three control variables: Psychological Resilience (PsyResil α = 0.73), Previous Work experience (PW, no = 0, yes = 1), and Independent Work experience (IW, no = 0, yes = 1), the last two being dichotomous. Resilience has been seen as a crucial, decisive and the trigger factor for the entrepreneurs’ success (e.g., Hedner et al., 2011). According to Rutter (2012), Psychological Resilience is considered an interactive process between the person and the social environment, which allow to renew themselves and dedicate themselves to success. In addition to being an important quality, it is a predictor of business success at all stages of the entrepreneurial process (Bullough and Renko, 2013; Cheng et al., 2020). Considering the last two variables, the literature points out that one of the factors that promote entrepreneurial intention is previous work experience (Carvalho and González, 2006).

### Statistical Procedure

To analyze the proposed model and to measure causal relationships, Structural Equation Modeling was used. For that, we used IBM SPSS Amos 23 and IBM SPSS 23 for the remaining analyzes (correlations, descriptive, and mean comparison).

According to Kline (2011), when the sample is greater than 200, the following indices are used: the Comparative Fit Index (CFI > 0.90), the Adjusted Goodness of Fit (GFI > 0.95) (Hair et al., 2010); the Root Square Error of Approximation (RMSEA < 0.05), the Tucker–Lewis Index (TLI > 0.90) (Awang, 2012), and the Expected Cross Validation Index (ECVI). This last index does not have specific threshold indexes, it is assumed that the lower the index, the better the fit and the better the model can predict the future covariance of the sample (Browne and Cudeck, 1992). Lastly, multiple squared correlations (R2) were made to demonstrate how much of the variation in the independent variables is explained by the predictors.

To calculate the coefficient and significance of the direct effects, the maximum likelihood estimate was used. To estimate mediation effects and group differences, it was Bootstrap with 2,000 interactions and 0.95 bias correction (Davidson and Hinkley, 1997). It was considered the product or the difference between the unstandardized regression weights, on the mediation or moderation path, to test whether the effect between the variables is statistically significant, at a 95% confidence level. The alpha was *p* < 0.05 for statistical significance.

The *t*-test statistic was used to calculate and compare the mean difference between both countries. In order to observe the homogeneity of the variables (>0.05), we used the Levene test.

## Results

### Model Fit

In this study we found the following model adjustment indexes for SEM: CFI = 0.992; TLI = 0.922; GFI = 0.976; RSME = 0.032 e; ECVI = 0.455. These results reveal a good fit of the model and above the common standards (Browne and Cudeck, 1992; Hair et al., 2010; Awang, 2012). Regarding the variance of the dependent variable, the R2 explains in the group of Spanish students 52% and in the group of Portuguese students 76%, which reveals that the values are adequate. In Table 2, it is possible to identify Pearson’s correlations, of which we highlight a strong and significant correlation between PBC and SEI. In this way, the results achieved allow us to recognize the necessary theoretical coherence, so we proceed to test the remaining hypotheses. Hence, we proceed with the analysis of the remaining hypotheses.

**TABLE 2:** Correlations analysis.

	**1**	**2**	**3**	**4**	**5**	**6**	**7**	**8**	**9**
(1) PBC	1								
(2) AT	0.711**	1							
(3) SN	0.522**	0.509**	1						
(4) Spirit	0.417**	0.148**	0.281**	1					
(5) Opt	0.327**	0.139**	0.126**	0.256**	1				
(6) PsyResil	0.352*	0.507**	0.605**	0.267**	0.116**	1			
(7) PW	0.004	0.184**	0.079*	0.082*	0.083**	0.121*	1		
(8) IW	0.032	0.199**	0.087**	0.117**	0.087**	0.024	0.116*	1	
(9) SEI	0.634**	0.673**	0.143**	0.294**	0.334**	0.250**	0.156*	0.122**	1

**Correlation is significant at the 0.01 level (two-tailed). *Correlation is significant at the 0.05 level (two-tailed), Pearson correlation.

### Regression Weights

To understand how each variable interacts it is important to highlight the weight of each regression, before elaborating the path model. Table 3 shows the values for both countries. The dimensions of the Theory of Planned Behavior (AT, PBC, and SN), the exogenous variables of the proposed model, significantly predict the SEI of university students. The PBC, for instance, has a stronger regression value, for both countries.

**TABLE 3:** Regression weights by country.

	**Spain**			**Portugal**		
	***B***	***SE***	***p***	***B***	***SE***	***p***
Opt ← PW	0.147	0.262	0.429	0.364	0.389	0.278
Opt ← IW	0.044	0.149	0.533	0.124	0.066	0.254
Opt ← AT	0.251	0.063	0.004**	0.169	0.043	***
Opt ← PBC	0.199	0.054	0.002**	0.078	0.031	0.066
Opt ← SN	–0.019	0.056	0.754	0.174	0.039	0.021*
Spirit ← PBC	0.059	0.069	0.627	0.189	0.038	***
Spirit ← AT	0.211	0.065	0.198	0.149	0.041	***
Spirit ← SN	0.322	0.074	0.148	0.152	0.042	0.021*
Spirit ← PsyResil	0.362	0.075	***	0.154	0.042	***
Spirit ← Opt	0.169	0.058	0.021*	0.229	0.071	***
Spirit ← IWE	0.051	0.312	0.891	-0.244	0.497	0.359
SEI ← PBC	0.642	0.068	***	0.528	0.052	***
SEI ← AT	0.421	0.082	***	0.126	0.038	0.004**
SEI ← SN	0.172	0.089	0.032*	0.328	0.054	***
SEI ← PW	–0.146	0.225	0.211	–0.072	0.054	0.608
SEI ← IW	0.082	0.394	0.630	–0.189	0.552	0.644
SEI ← PsyResil	0.378	0.072	0.022*	0.114	0.041	0.036*
SEI ← Opt	0.148	0.081	0.112	0.287	0.046	***
SEI ← Spirit	0.388	0.040	0.239	0.071	0.089	***

B: unstandardized estimates; ***p = 0.001 or less; is significant at the <0.05 value, **p < 0.01; *p < 0.05. Maximum likelihood estimation.

There is a significant difference between coefficients from the other exogenous variables: for the Spanish student’s group, *p* = 0.016 when compared to AT, and *p* = 0.001 when compared to SN. Regarding the group of Portuguese students, *p* = 0.001 when compared to AT, and *p* = 0.001 when compared to SN. However, Spanish students reach higher coefficients on PBC, and AT; and students from Portugal reach higher on SN. That is, the perception of these variables by students from both countries affects their intentions, although not significantly different from each other. For Spanish students, AT and PBC have a statistically significant regression to Optimism. However, neither impact significantly the Spirituality. Regarding students from Portugal, PBC has not a significant regression in the Optimism, and in the case of Spirituality both (PBC and AT) have a significant regression. SN has a significant impact in students from Portugal for Optimism, but the same is not verified in the group of Spanish students.

Spirituality impacts significantly SEIs in Portuguese students, but not in students from Spain. Similarly, Optimism effect is drastically stronger and significant on SEI in students from Portugal, but not Spanish students.

Regarding the other control variables, PsyResil effect is positive and significant on SEIs and on Spirituality in both countries. Interactions with PW and IW presented non-significant and/or negative effects. Both PW and IW impact negatively in Portuguese students’ SEI; in the case of students from Spain, only PW has a negative effect on SEI.

### Path Model Effects

Table 4 presents the results obtained from our path model by Spanish and Portuguese students. When students with a favorable and elevated perception to achieve an entrepreneurial behavior of a social nature, this increases their Optimism and the intention to create a social entrepreneurial activity. Hence, the Optimism mediates a very positive and significant effect between PBC and SEI in Portuguese students. The relationship between SN and SEI is also positively and significantly mediated by Optimism, but only in Spanish students.

**TABLE 4:** Effects for path model by country.

	**Spain**				**Portugal**			
	**Effects**		**CI**		**Effects**		**CI**	
	***B***	***p***	**Lower**	**Upper**	***B***	***p***	**Lower**	**Upper**
	0.598	***	–	–	0.497	***	–	–
AT → SEI	0.229	0.006**	–	–	0.111	0.004*	–	–
SN → SEI	0.143	0.042*	–	–	0.178	***	–	–
PBC → Spirit → SEI	0.003	0.625	–0.012	0.026	0.106	0.003**	0.011	0.059
AT → Spirit → SEI	0.008	0.373	–0.012	0.063	0.017	0.027*	0.004	0.038
SN → Spirit → SEI	0.011	0.278	–0.014	0.076	0.145	0.003**	0.012	0.098
PBC → Opt → SEI	0.031	0.048*	0.000	0.086	0.055	***	0.012	0.069
AT → Opt → Spirit	0.055	0.077	–0.003	0.099	0.028	0.022	0.001	0.046
SN → Opt → SEI	0.018	0.015*	0.005	0.041	–0.004	0.0719	–0.018	0.024
PBC → Opt → Spirit	0.036	0.021*	0.007	0.098	0.014	0.056	0.002	0.036
AT → Opt → Spirit	0.041	0.037*	0.005	0.111	0.035	***	0.013	0.059
Opt → Spirit → SEI	0.011	0.358	–0.018	0.060	0.021	0.026*	0.005	0.051
PBC → Opt → Spirit → SEI	0.005	0.275	–0.003	0.014	0.003	0.052	0.000	0.004
AT → Opt → Spirit → SEI	0.005	0.332	–0.006	0.019	0.005	0.016*	0.001	0.011

B: standardized estimates; ***p = 0.001 or less; *p* is significant at the <0.05 value. **p < 0.01; ^∗^*p* < 0.05. Indirect effects: Bootstrapping: 2000 iterations and 0.95 bias-corrected; Direct effects: Maximum Likelihood Estimation.

Spirituality mediates a significant effect between PBC and SEI in Portuguese students, but not in students from Spain. This variable also mediates a significant effect between AT and SEI, and between SN and SEI in students from Portugal, but not in Spanish students. Finally, in this study we ran a serial mediation path and found that AT positively affects Optimism, which affects Spirituality and that in turn affects SEI, only in students from Portugal. These findings highlighted that AT is a determining factor for Portuguese students to achieve their entrepreneurial activities, here in particular, those of a social nature.

### Mean Comparison Between Countries

The biggest responses’ difference we highlighted concerns Optimism, with a mean difference of 0.313 (significant, *p* < 0.001); and the smallest differences concerns Subjective Norm, with a value of mean difference of 0.034 (not significant, *p* < 0 0.156). As countries in southern Europe, and classified as peripheral countries, Portugal and Spain faced similar challenges and difficulties during the economic crisis (Fernandes et al., 2013). However, regardless of the nationality and cultural background of university students, it is possible to conclude that the fact there is no difference between them and this is due to easy access to resources and also to government programs. These resources can concern several areas, such as financial support, the promotion and improvement of skills and competences, both essential for a young adult to be able to take the first steps as an entrepreneur.

Table 5 demonstrates the mean of each variable by country, and the results obtained in *t*-test analysis.

**TABLE 5:** Country means and *t*-test.

**Variable**	**Mean by country**				***t*-test for Equality of means**		
	**Country**	**Mean**	***SD***	***SE***	***t***	***p***	**Mean dif.**
PBC	Spain	3.816	0.802	0.026	–2.329	0.018	–0.180
	Portugal	3.996	0.753	0.032			
AT	Spain	3.579	0.736	0.025	–0.889	0.517	–0.38
	Portugal	3.959	0.867	0.034			
SN	Spain	3.712	0.725	0.025	2.781	0.156	0.034
	Portugal	3. 678	0.915	0.036			
Spirit	Spain	4.608	3.036	0.105	–3.161	0.001	–0.272
	Portugal	4.880	2.859	0.113			
Optimism	Spain	3.704	0.463	0.016	0.489	0.001	0.313
	Portugal	3.391	0.496	0.019			
SEI	Spain	3.174	0.387	0.013	–2.448	0.001	–0.283
	Portugal	3.457	0.544	0.021			

*Mean difference positive value means males score higher, negative value means females score higher. For *t*–test = Equality of variance is assumed in all variables: Levene’s Test = *p* > 0.05; 95% Confidence Interval for lower and upper values.*

## Discussion of Results

The first social group of the individual, the family, is identified as the most determinant for the development of entrepreneurial behavior (Bohnenberger et al., 2007; GEM, 2020). Other authors (e.g., Colette, 2013; Boldureanu et al., 2020) make it clear that entrepreneurial behavior can be learned and entrepreneurship education programs can positively influence students’ entrepreneurial intention. Hence, it is important to highlight the relevance of academic institutions for improving the perception of the social benefits of entrepreneurship (Barba-Sánchez and Atienza-Sahuquillo, 2017; Boldureanu et al., 2020). Moreover, youth entrepreneurship support organizations play a leading role in promoting these behaviors among the younger strata. In Portugal, it is possible to highlight the National Association of Young Entrepreneurs, which works to promote incentive systems, business advice to young entrepreneurs, incubate start-ups and support the internationalization of companies. In Spain, Junior Achievement works with schools, business organizations and governments to provide young people with experiences that help them develop the skills and competencies needed to succeed in a global economy through entrepreneurial activities.

As a result of European, Portuguese, and Spanish directives, entrepreneurial activity has increasingly proved to be one of the privileged channels for ’making the leap’ into the labor market (European Commission, 2013; Marques, 2015; Hervas-Oliver et al., 2017). Several studies indicate that are the students of business and economics who are more likely to start their own company, once they have higher levels of entrepreneurial intention (e.g., Sieger et al., 2014). However, Portuguese (Santos et al., 2013) and Spanish students (Ward et al., 2019) of Social Sciences are the ones who expressed the most intention to undertake. Another study referring to students from Portugal reveals that are the Pharmacy students who reveal to have more entrepreneurial characteristics (Teixeira, 2008). Therefore, it is important to reflect on the University’s role as a means of promotion and education for entrepreneurship among students. The student’s field of studies has proved to be as one of the least relevant when evaluating their intentions. In the educational university environment, learning for entrepreneurship aims to help building the spirit, skills and entrepreneurial culture of students (Hasan et al., 2017). According to Ward et al. (2019), it is evident that business students are not the only holders of entrepreneurial skills. According to another study, it is possible to identify that entrepreneurship skills are supported by appropriate learning programs within the educational institution (Hasan et al., 2017).

Spanish students’ perceived social pressure to perform or not a behavior is higher than students from Portugal. However, this variable presents as a predictor of SEI, which is not significantly different between both countries. Contrary to what is pointed out in the study with students from Spain by Miranda et al. (2017), which states that there was not enough empirical evidence to establish a significant relationship between SN and intentions to undertake. Many other studies contrast with the previous idea (e.g., Huyghe and Knockaert, 2015; Obschonka et al., 2015). The subjective norm is the most social component and, in turn, implies a person’s belief about the presence of social pressure to perform an action or not, and the motivation to satisfy this pressure. A positive relationship was found between subjective norms and SEI, which reflects the desirability of promoting the development of SE projects in the educational field, especially in university education, as in these ages the promotion of social motivation may have greater impact (Barba-Sánchez and Atienza-Sahuquillo, 2017; Ruiz-Rosa et al., 2020).

Optimism mediates the effect between subjective norm and intentions in students from Spain, but not in Portuguese students. This may suggest that optimistic students, with positive relationships with others and personal mastery will be more realistic and flexible, once it is a strong learning in terms of self-discipline, analysis of past mistakes, and planning to prevent the occurrence of negative events. This idea could be relevant under determining intentions and it may be a potential explanation for the influence (or lack thereof) in the intentions of both countries.

Portugal is presented as a country where the decision-making to start an entrepreneurial activity is stimulated by the social environment and positions 39 in a range of 190 economies with regard to the ease of setting up a company (Ease of Doing Business Index, 2020). The same source reveals that Spain appears better positioned with regard to ‘doing business,’ in the 30th position, and seeks to be a country that protects minority investors. Social recognition is a stronger indicator of SEI in Spain than in Portugal. In other words, despite the perception of the existence of support programs, the avoidance of uncertainties and taking risks in Portugal suggests that starting an entrepreneurial activity is considered an uncertain working path. Therefore, the decision-making to become an entrepreneur in Portugal is culturally less likely and acceptable. This result is in line with data from the António Sérgio Cooperative for Social Economy. Between March and June 2020, there was a decrease –60% in applications due to the pandemic crisis. Whereas in Spain, six out of ten entrepreneurs created a new company during the Covid-19 Crisis. The Observatory of Entrepreneurship of Spain also points out that 61% of the initiatives to create its own business continued its development process, during and after the pandemic crisis (GEM, 2020). These data allow us to conclude that the ability to identify new opportunities combined with resilience and innovation, in times of pandemic and uncertainty, are the reflection of an optimistic future that can prosper. In this sense, the pandemic is bringing more and more training and support programs to the entrepreneurial ecosystem. For instance, the School of Industrial Organization (EOI) in collaboration with Cisco Spain, co-financed by the European Social Fund and the Spanish Ministry of Industry, Commerce and Tourism promotes online courses for 100 unemployed women (European Commission, 2020).

The outcomes of this study revealed that PBC predicts significantly stronger than subjective norms on SEIs in both countries. And it did not have a significantly different effect between Spanish and Portuguese students. The PBC has shown a volatility in the empirical results related to its influence on the intention and also to a certain lack of agreement regarding the concept and operationalization (Yap et al., 2013). It is a concept that is associated with high self-efficacy and effectiveness (Zhao et al., 2010). The difference in the mean between both countries is statistically significant, however, the values reached are not high. It is important to mention that PBC concerns the self-assessment of the abilities/skills and knowledge of individuals regarding to the intention to start an enterprise (Heidemann et al., 2012; Fragoso et al., 2020). Hence, the importance of the university in setting up innovation environments and ensuring training spaces to enhance entrepreneurship (Carvalho et al., 2017; Fragoso et al., 2020).

The results for Spanish students revealed that the greater the level of agreement in which an individual perceives that business activities are favored in their different social circles, the greater will be their ability to resist an adverse situation, which would also affect their entrepreneurial social intention. Something similar happens with Optimism. This may mean that when individuals believe that their future holds positive outcomes, or that there is a positive side of every experience, allows them to define more clearly a path toward entrepreneurship. This is an interesting fact, because the study by Giacomin et al. (2016) found that the optimism was not significant for the intentions in Spanish students. The optimism (or pessimism) of the social entrepreneur’s network of important people can be a guideline for the future development of the idea of creating a social enterprise (Vasakarla, 2008). However, according to the same source, this network is not relevant to the final decision to create the social activity. It is important to mention that a highly optimistic individuals are persistent and tends to exhibit diffuse confidence, which allows them to face challenges with enthusiasm (Carver and Scheier, 2003). However, other research points out that high optimism can lead to false expectations and, therefore, to negative results (Gibson and Sanbonmatsu, 2004). And it can also have detrimental effects on judgment and decision-making.

According to Muscat and Whitty (2009), SE requires leadership based on ethics and spirituality, with the purpose of finding the common good, through sustainability. Yitshaki and Kropp (2016) go further and, in addition to the prosocial, family and life experience reasons, they link personal values and spiritual guidance as triggers of SE. Our findings indicate that spirituality mediates the relationship between PBC and SEI, and between attitude toward a behavior and intentions to undertake in a social way in Portuguese students. The way in which spirituality influences the individual to have a behavior that creates a positive effect on society and his/her perceived ability to overcome obstacles, makes them more resilient and persevering (Margaça et al., 2021). In addition, spirituality also empowers people to face the adversities of daily life, in a personal and unique way (Benavent, 2014). That is, the dynamics of values intrinsic to spirituality, such as compassion, empathy, dignity and solidarity (Ghandi and Raina, 2018) can encourage social entrepreneurial activity (Chandra and Shang, 2017). Social entrepreneurs are seen as the catalysts of society, through opportunities that change society for the better. Roundy et al. (2016) pointed to the union of their religious and/or spiritual beliefs and work as one of the drivers to create an entrepreneurial activity of a social nature.

This article has implications for educators, policy makers, researchers, university students and potential entrepreneurs. It will also act as a comparison across two different cultures, allowing for robust testing of a model that can help explain SEIs. The results can be useful for policy makers to understand not only the pattern of antecedents of intention, but also their implications for their interventions, namely in terms of financial support.

## Theoretical Contributions

This research contributed to the literature on (social) entrepreneurial intention by developing and testing a model in two countries, considering the role of culture and psychological variables. The literature points to a path where SE began to be explored at various levels, such as reaching investors (e.g., Roundy, 2017) or innovating in their business model (e.g., Mair and Shoen, 2007). However, very little is known about the true motives that lead the individual to undertake for a social cause. In general, this study contributes to the literature on entrepreneurship and, in particular, to SE, by creating causal relationships between two psychological resources.

This study also made it possible to understand how soft skills, such as optimism, and spirituality as a core value influence the decision-making process to create a social entrepreneurial activity. The inclusion of spirituality concept in a model of SEI provides a deeper understanding of this process and the variations between two different societies culturally different. This fact also highlights possible factors and personal values to be considered in the development of more comprehensive models regarding the social aspect of entrepreneurship. From social causes come the desire to serve the common good, for instance, authors like Gjorevska (2019) presents examples of entrepreneurship driven by spirituality and how spirituality can be one of the pro-social reasons.

As several studies on entrepreneurial intentions point out (e.g., Ward et al., 2019; Margaça et al., 2021), also when we study the intention to engage in SE, the individuals’ training/academic area is not limited to the area of management and business. Few studies have been done on the relationship between variables such as spirituality and its intrinsic values and SEI. Hence, we believe that, on the one hand, studies on the intention to undertake socially should encompass all academic areas and, on the other hand, start to attribute more meaning to personal values and beliefs, in order to complement entrepreneurship education programs.

## Practical Implications

Nowadays, SE is a concept that enables business consolidation, combined with the production of positive impact and improvements in society. This finding reinforces the importance of the role of universities in improving the offer of programs that foster entrepreneurship. It is important to mention that universities that lead rankings such Times Higher Education, offer courses in SE (aimed at those who intend to develop or improve projects), or courses in management of non-governmental and non-profit organizations. Thus, policy makers and institutions responsible for creating entrepreneurship training programs, should pay more attention and bring values and methods of SE to traditional training and education. It would also be interesting, as highlighted by Huyghe and Knockaert (2015), to incorporate new incentive systems for academics. That is, changing the individual’s perspective in the sense of not only looking at his teaching and research performance, but also encouraging the transfer of results, for example, through patent licensing, creation of spin-offs.

Social enterprises play an important role in tackling societal challenges (Misuraca et al., 2016). Given the profile of the social entrepreneur (Sastre-Castillo et al., 2015), it is important to consider the emotional aspects and personal values. Through testimonials, career planning workshops and, through design thinking method, it is possible to equip the individual with skills to deal with resulting difficulties, allowing them to develop their self-efficacy (González-López et al., 2019). The longitudinal monitoring of these programs is crucial, ensuring a real evaluation of the results. In addition, it is important to generate new solutions to social problems, boosting the social investment market, creating more adequate financing instruments and, above all, training the actors of the social innovation and entrepreneurship system. Fortunately, there are several entities in Portugal and Spain that offer support and financing solutions to social projects. Portugal Social Innovation is a public initiative that aims to promote social innovation and boost the social investment market in the country. Creas is a hands-on investor that, in addition to financing, provides active support to the management team of its investees and access to a network of top-level professionals. Pioneers in Spain in using impact investing, Creas fosters a business model that creates social value and transforms reality toward a world where the human being and the planet are at the center of decision-making.

From a practical and professional point of view, our results can be particularly useful in the design of SE courses and in the respective selection of participants. We consider it to be a positive implication, as the selection of candidates based on psychological criteria can contribute to a better career suitability and, thus, increase the number of social entrepreneurs (Kruse et al., 2020; Nakao and Nishide, 2020).

Finally, due to the 2030 Agenda for the achievement of the United Nations Sustainable Development Goals, a new era of collaborative SE with a focus on promoting large-scale systemic change is emerging. It is important to clarify the role of social entrepreneurs, as they are making visible the impact of their creative ideas in several areas, from civic engagement to the environment, health and learning. Thus, from global perspective, our findings bring new insights into the need for synergy between governments, civil society and social entrepreneurs to pay close attention to the enormous social changes we are facing.

## Limitations and Suggestions for Future Research

The present study has some limitations that should be addressed in future studies. The variables used in the study allowed us to assess students’ perception and intentionality regarding SE. Although we believe that the outputs are positive and promising, it is important to introduce other variables and theories that allow us to draw a more consistent social entrepreneur profile. A methodological limitation is related to the fact that the PBC construct is better evaluated through the variables perceived controllability and perceived self-efficacy (Vamvaka et al., 2020). In order to access more trusted results, these two variables must be used in the future.

Therefore, it becomes extremely important to study a completer and more robust model, for instance, where the evaluation extends to characteristics such as sustainability and innovation, in parallel with the Theory of Self-determination. Regarding the sample, we consider that in the future it may be extended to other countries with greater cultural differences, which would allow a broader range of results. It would also be important to consider a gender-equal sample. Another issue is related to the fact that the sample is composed of students. For a better evaluation it would be important to consider a longitudinal research to evaluate the effectiveness and the materialization of their intentions.

## Data Availability Statement

The raw data supporting the conclusions of this article will be made available by the authors, without undue reservation.

## Author Contributions

CM: writing, sampling, statistics and discussion. BH-S: writing and discussion. GC: discussion. JS-G: discussion. All authors: contributed to the article and approved the submitted version.

## Conflict of Interest

The authors declare that the research was conducted in the absence of any commercial or financial relationships that could be construed as a potential conflict of interest.

## References

[B1] Ahmed I. Nawaz M. Ahmad Z. Shaukat M. Usman A. Rehman W. (2010). Determinants of students’ entrepreneurial career intentions: evidence from business graduates. *Euro. J. Soc. Sci.* 15 14–22.

[B2] Ahuja V. Akhtar A. Wali O. (2019). Development of a comprehensive model of social entrepreneurial intention formation using a quality tool. *J. Global Entrep. Res.* 9 1–27. 10.1186/s40497-019-0164-4

[B3] Ajzen I. (1991). The Theory of Planned Behavior. *Org. Behav. Human Dec. Proc.* 50 179–211.

[B4] Ajzen I. (2002). Perceived Behavioral control, self-efficacy, locus of control, and the theory of planned behavior. *J. Appl. Soc. Psychol.* 32 665–683. 10.1111/j.1559-1816.2002.tb00236.x

[B5] Amit R. MacCrimmon K. Zietsma C. Oesch J. (2001). Does money matter? Wealth attainment as the motive for initiating growth-oriented technology ventures. *J. Bus. Vent.* 16 119–143. 10.1016/S0883-9026(99)00044-0

[B6] Austin J. E. Stevenson H. Wei-Skillern J. (2006). Social and commercial entrepreneurship: Same, different, or both. *Entrep. Theory Pract.* 30 1–22. 10.1111/j.1540-6520.2006.00107.x

[B7] Autio E. Keeley R. H. Klofsten M. Parker G. Hay M. (2001). Entrepreneurial Intent Among Students in Scandinavia and in the USA. *Enterp. Innov. Manag.* 2 145–160. 10.1080/14632440110094632

[B8] Awang Z. (2012). *Structural Equation Modeling Using AMOS Graphic.* Malaysia: Universiti Teknologi MARA Press.

[B9] Balog A. Baker T. Walker A. (2014). Religiosity and spirituality in entrepreneurship: a review and research agenda. *J. Manag. Spirit. Relig.* 11 159–186. 10.1080/14766086.2013.836127

[B10] Barba-Sánchez V. Atienza-Sahuquillo C. (2017). Entrepreneurial motivation and self-employment: evidence from expectancy theory. *Int Entrep Manag J* 13 1097–1115. 10.1007/s11365-017-0441-z

[B11] Benavent E. (2014). Espiritualidad: heterodoxia y punto de encuentro, un activo para la educación social. *Rev. Interv. Socioeduc.* 56 13–30.

[B12] Bird B. (1988). Implementing entrepreneurial ideas: the case for intention. *Acad. Manag. Rev.* 13 442–453. 10.2307/258091

[B13] Bird B. J. Schjoedt L. (2009). “Entrepreneurial behavior: its nature, scope, recent research and agenda for future research,” in *Understanding the entrepreneurial mind: opening the black box*, eds Carsrud A. Brannback M. (Berlin: Springer Science and Business Media, LLC), 327–358. 10.1007/978-1-4419-0443-0_15

[B14] Bohnenberger M. Schmidt S. Freitas E. (2007). “A influência da família na formação empreendedora,” in *Paper Presented at XXXI Encontro da ANPAD*, (Brazil).

[B15] Boldureanu G. Ionescu A. Bercu A. Bedrule-Grigoruta M. Boldureanu D. (2020). Entrepreneurship Education through Successful Entrepreneurial Models in Higher Education Institutions. *Sust.* 12 10.3390/su12031267

[B16] Bornstein D. (2004). *How to change the world: social entrepreneurs and the power of new ideas.* Oxford: Oxford University Press.

[B17] Bornstein D. Davis S. (2010). *Social entrepreneurship - What everyone needs to know.* Oxford: Oxford University Press.

[B18] Bosma N. Schøtt T. Terjesen S. Kew P. (2016). *Global Entrepreneurship Monitor 2015 to 2016: Special Report on Social Entrepreneurship.* Global Entrepreneurship Research Association. Available at SSRN: https://ssrn.com/abstract=2786949 or 10.2139/ssrn.2786949

[B19] Browne M. W. Cudeck R. (1992). Alternative ways of assessing model fit. *Sociol. Methods Res.* 21 230–258. 10.1177/0049124192021002005

[B20] Bullough A. Renko M. (2013). Entrepreneurial resilience during challenging times. *Bus. Horiz.* 56 343–350. 10.1016/j.bushor.2013.01.001

[B21] Bullough A. Renko M. Myatt T. (2014). Danger zone entrepreneurs: the importance of resilience and self-efficacy for entrepreneurial intentions. *Entrep. Theory Pract.* 38 473–499. 10.1111/etap.12006

[B22] Candland C. (2000). Faith as social capital: religion and community development in Southern Asia. *Pol. Scie.* 33 355–374.

[B23] Carvalho P. González L. (2006). Modelo explicativo sobre a intenção empreendedora. *Comport Organ. Gest.* 12 43–65.

[B24] Carvalho S. Aveni A. Coimbra L. Montilha H. (2017). Empreendedorismo, Tecnologia e Inovação: temas contemporâneos na gestão da Universidade de Brasília. *Cad. Prospec.* 10 626–638. 10.9771/cp.v10i4.23017

[B25] Carver C. S. Scheier M. (2003). “Optimism,” in *Positive psychological assessment: A handbook of models and measures*, eds Lopez S. J. Snyder C. R. (Washington DC: American Psychological Association), 75–89.

[B26] Casaqui V. (2014). Concepções e significados do empreendedorismo social no Brasil e em Portugal: crise, performance e bem comum. *Observat.* 8 67–82. 10.15847/obsOBS822014701

[B27] Chandra Y. Shang L. (2017). Social Enterprise as a mechanism of youth empowerment. *Hong Kong J. Soc. Work* 51 10.1142/S0219246217000080

[B28] Chapman T. Chi T. (2017). Perceived Social Support mediates the link between optimism and active coping. *J. Behav. Soc. Sci.* 4 57–65.

[B29] Cheng S. King D. Oswald F. (2020). Understanding how resilience is measured in the organizational sciences. *Hum. Perform.* 33 130–163. 10.1080/08959285.2020.1744151

[B30] Cohen B. Smith B. Mitchell R. (2008). Toward a sustainable conceptualization of dependent variables in entrepreneurship research. *Bus. Strat. Env.* 17 107–119. 10.1002/bse.505

[B31] Colette H. (2013). Entrepreneurship education in H.E.: are policy makers expecting too much? *Educ. Train.* 55 836–848. 10.1108/et-06-2013-0079

[B32] Collins S. (2007). Statutory social workers, job satisfaction, coping, social support and individual differences. *British J. Soc. Work* 38 1173–1193. 10.1093/bjsw/bcm047

[B33] Dana L. P. (2010). *Entrepreneurship and religion.* Cheltenham, USA: Edward Elgar Publishing.

[B34] Dana L. P. Wright R. (2004). “Emerging paradigms of international entrepreneurship,” in *Handbook of Research on International Entrepreneurship*, ed. Dana L. P. (Northampton: Edward Elgar Publishing), 3–15.

[B35] Davidson A. Hinkley D. (1997). *Bootstrap Methods and their application.* Cambridge: Press Sindicate of the University of Cambridge.

[B36] Devece C. Peris-Ortiz M. Rueda-Armengot C. (2016). Entrepreneurship during economic crisis: success factors and paths to failure. *J. Bus. Res.* 69 5366–5370. 10.1016/j.jbusres.2016.04.139

[B37] Dushnitsky G. (2010). Entrepreneurial optimism in the market for technological inventions. *Org. Scie.* 21 150–167. 10.1287/orsc.1090.0454 19642375

[B38] Ease of Doing Business Index (2020). *International Bank for Reconstruction and Development.* Washington: Library of Congress.

[B39] Elfving J. (2008). *Contextualizing Entrepreneurial Intentions: a Multiple Case Study on Entrepreneurial Cognition and Perception.* Turku: Åbo Akademi Förlag.

[B40] Ernst K. (2011). *Heart over mind – An empirical analysis of social entrepreneurial intention formation on the basis of the theory of planned behavior. Doctoral Thesis.* Berlin, Germany: University of Wuppertal, 1–309. Available at http://nbn-resolving.de/urn/resolver.pl?urn=urn:nbn:de:hbz:468-20120327-142543-6

[B41] European Commission (2013). *Entrepreneurship 2020 Action Plan: Reigniting the Entrepreneurial Spirit in Europe.* Brussels: Available at http://eurlex.europa.eu/LexUriServ/LexUriServ.do?uri=COM:2012:0795:FIN:en:PDF (accessed January 10, 2021).

[B42] European Commission (2020). *European Social Fund.* Brussels: Available at https://ec.europa.eu/social/main.jsp?langId=en&catId=325 (accessed January 10, 2021).

[B43] Fernandes I. Ferreira J. Raposo M. (2013). Drivers to firm innovation and their effects on performance: an international comparison. *Int. Entrep. Manag. J.* 9 557–580. 10.1007/s11365-013-0263-6

[B44] Fernando M. (2007). *Spiritual leadership in entrepreneurial business: a multifaith study.* Northampton, MA: Edward Elgar Publishing, Inc.

[B45] Fini R. Grimaldi R. Marzocchi G. L. Sobrero M. (2012). The determinants of corporate entrepreneurial intention within small and newly established firms. *Entrep. Theory Pract.* 36 387–414. 10.1111/j.1540-6520.2010.00411.x

[B46] Forster F. Grichnik D. (2013). Social Entrepreneurial Intention Formation of Corporate Volunteers. *J. Soc. Entrep.* 4 153–181. 10.1080/19420676.2013.777358

[B47] Fragoso R. Rocha-Junior W. Xavier A. (2020). Determinant factors of entrepreneurial intentions among universiy students in Brazil and Portugal. *J. Small Bus. Entrep.* 32 33–57. 10.1080/08276331.2018.1551459

[B48] GEM (2020). *Global Entrepreneurship Monitor 2019/2020 Global Report.* London: Global Entrepreneurship Research Association/London Business School.

[B49] Ghandi T. Raina R. (2018). Social entrepreneurship: the need, relevance, facets and constraints. *J. Global Entrep.Res.* 8

[B50] Giacomin O. Janssen F. Shinnar R. S. (2016). Student entrepreneurial optimism and overconfidence across cultures. *Int. Small Bus. J.* 34 925–947. 10.1177/0266242616630356

[B51] Gibson B. Sanbonmatsu D. M. (2004). Optimism, pessimism, and gambling: The downside of optimism. *Pers. Soc. Psyc. Bullet.* 30 149–160. 10.1177/0146167203259929 15030630

[B52] Gjorevska N. (2019). “Workplace Spirituality in Social Entrepreneurship: Motivation for Serving the Common Good,” in *Servant Leadership, Social Entrepreneurship and the Will to Serve: Spiritual Foundations and Business Applications*, eds Bouckaert L. van den Heuvel S. (London: Palgrave Macmillan), 187–209. 10.1007/978-3-030-29936-1_10

[B53] González-López M. Pérez-López M. Rodríguez-Ariza L. (2019). Clearing the hurdles in the Entrepreneurial race: the role of resilience in entrepreneurship education. *Acad. Manag. Learn. Edu.* 18 457–483. 10.5465/amle.2016.0377

[B54] Grégoire D. Corbett A. McMullen J. (2011). The cognitive perspective in Entrepreneurship: an agenda for future research. *J. Manag. Stud.* 48 143–1477. 10.1111/j.1467-6486.2010.00922.x

[B55] Hair J. Black W. Babin B. Anderson R. (2010). *Multivariate Data Analysis.* Upper Saddle River, NJ: Prentice-Hall, Inc.

[B56] Hasan M. Khan E. A. Nabi N. (2017). Entrepreneurial education at university level and entrepreneurship development. *Educ. Train.* 59 888–906. 10.1108/ET-01-2016.0020

[B57] Hedner T. Abouzeedan A. Klofsten M. (2011). Entrepreneurial resilience. *Ann. Innov. Entrep.* 2 7986. 10.3402/aie.v2i1.6002

[B58] Heidemann L. Araújo I. Veit E. (2012). Um referencial teórico-metodológico para o desenvolvimento de pesquisas sobre atitude: a Teoria do Comportamento Planejado de Icek Ajzen. *Rev. Elet. Inv. Educ. Cienc.* 7 22–31.

[B59] Hervas-Oliver J. Boronat-Moll C. Mesana-Salinas I. (2017). La universidad española como plataforma de emprendimiento: hacia la universidad emprendedora del futuro. *Eco. Ind.* 404 11–19.

[B60] Hockerts K. (2017). Determinants of social entrepreneurial intentions. *Entrep.Theory Pract.* 41 105–130. 10.1111/etap.12171

[B61] Hodge D. R. (2003). The intrinsic spirituality scale. *J. Soc. Serv. Res.* 30 41–61. 10.1300/J079v30n01_03

[B62] Huyghe A. Knockaert M. (2015). The influence of organizational culture and climate on entrepreneurial intentions among research scientists. *J. Tech. Transf.* 40 138–160. 10.1007/s10961-014-9333-3

[B63] Instituto Nacional de Estadística [INE] (2013). *Tasas de paro por distintos grupos de edad, sexo y comunidad autónoma.* Instituto Nacional de Estadística. Available at https://www.ine.es/jaxiT3/Tabla.htm?t=4247 (accessed December 27, 2020).

[B64] Irengün O. Arikboga S. (2015). The effect of personality traits on social entrepreneurship intentions: a field research. *Procedia Soc. Behav. Scie.* 195 1186–1195. 10.1016/j.sbspro.2015.06.172

[B65] Katz J. A. (1992). A psychological cognitive model of employment status choice. *Entrep. Theory Pract.* 17 29–37. 10.1177/104225879201700104

[B66] Kauanui S. Thomas K. Rubens A. Sherman C. (2010). Entrepreneurship and Spirituality: a comparative analysis of entrepreneurs’ motivation. *J. Small Bus. Entrep.* 23 621–635. 10.1080/08276331.2010.10593505

[B67] Kinjerski V. Skrypnek B. (2004). Defining spirit at work: finding common ground. *J. Org. Change Mang.* 17 26–42. 10.1108/09534810410511288

[B68] Kline R. (2011). *Principles and Practice of Structural Equation Modeling.* New York: Guilford.

[B69] Kolvereid L. Isaksen E. (2006). New business start-up and subsequent entry into self-employment. *J. Bus. Vent.* 21 866–885. 10.1016/j.jbusvent.2005.06.008

[B70] Krueger N. (2009). “Entrepreneurial Intentions are Dead: Long Live Entrepreneurial Intentions,” in *Understanding the Entrepreneurial Mind*, eds Carsrud A. Brännback M. (Berlin: Springer), 51–72. 10.1007/978-1-4419-0443-0_4

[B71] Krueger N. Brazeal D. (2017). Potencial empreendedor e empreendedores em potencial. *Rev. Empreend. Gest. Peq. Emp.* 7 201–226. 10.14211/regepe.v7i2.1071

[B72] Krueger N. Schulte W. Stamp J. Kickul J. (2007). “Beyond intent: precipitating events for social entrepreneurial intentions,” in *Communication presented at the 3rd International Social Entrepreneurship Research Conference (ISERC3)*, (Copenhagen).

[B73] Krueger N. F. Reilly M. D. Carsrud A. L. (2000). Competing Models of Entrepreneurial Intentions. *J. Bus. Vent.* 15 411–432. 10.1016/S0883-9026(98)00033-0

[B74] Kruse P. Wach D. Wegge J. (2020). What motivates social entrepreneurs? A meta- analysis on predictors of the intention to found a social enterprise. *J. Small Bus. Manag.* 1–32. 10.1080/00472778.2020.1844493

[B75] Li C. Wu J. (2011). The structural relationships between optimism and innovative behavior. *Creat. Res. J.* 23 119–128. 10.1080/10400419.2011.571184

[B76] Liñán F. (2008). Skill and Value Perceptions: How Do they Affect Entrepreneurial Intentions? *Int. Entrep. Manag. J.* 4 257–272. 10.1007/s11365-008-0093-0

[B77] Liñán F. Chen Y. W. (2009). Development and cross-cultural application of a specific instrument to measure entrepreneurial intentions. *Entrep. Theory Pract.* 33 593–617. 10.1111/j.1540-6520.2009.00318.x

[B78] Liñán F. Nabi G. Krueger N. (2013). British and Spanish entrepreneurial intentions: a comparative study. *Rev. Econ. Mund.* 33 73–103.

[B79] Liñán F. Urbano D. Guerrero M. (2011). Regional variations in entrepreneurial cognitions: start-up intentions of university students in Spain. *Entrep. Reg. Develop.* 23 87–215. 10.1080/08985620903233929

[B80] Lough B. J. McBride A. M. (2013). The influence of solution-focused reflection on international social entrepreneurship identification. *J Social Entrep.* 4 220–236. 10.1080/19420676.2013.777361

[B81] Madari N. Teeni-Harari T. Icekson T. Sela Y. (2019). Optimism and entrepreneurial intentions among students: the mediating role of emotional intelligence. *J. Entrep. Educ.* 22 1–19.

[B82] Maes J. Leroy H. Sels L. (2014). Gender differences in entrepreneurial intentions: A TPB multi-group analysis at factor and indicator level. *Euro. Manag. J.* 32 784–794. 10.1016/j.emj.2014.01.001

[B83] Mair J. Noboa E. (2005). How intentions to create a social venture are formed: A case study. *SSRN Elect. J.* 3 1–29. 10.2139/ssrn.875589

[B84] Mair J. Noboa E. (2006). “Social entrepreneurship: how intentions to create a social enterprise get formed,” in *Social Entrepreneurship*, eds Mair J. Hockerts K. (London: Palgrave), 121–135. 10.1057/9780230625655_8

[B85] Mair J. Shoen O. (2007). Successful social entrepreneurial business models in the context of developing economies: an explorative study. *Int. J. Emerg. Mark.* 2 54–68. 10.1108/17468800710718895

[B86] Margaça C. Hernández-Sánchez B. Sánchez-García J. C. Cardella G. M. (2021). The Roles of Psychological Capital and Gender in University Students’ Entrepreneurial Intentions. *Front. Psyc.* 11 10.3389/fpsyg.2020.615910 33519639 PMC7843387

[B87] Marques A. (2015). Entrepreneurial learning in higher education: perceptions, realities and collaborative work from the stakeholder point of view. *Eur. J. Soc. Sci. Edu. Res.* 5 254–261.

[B88] Mehrabian A. Epstein N. (1972). A measure of emotional empathy. *J. Pers.* 40 525–543. 10.1111/j.1467-6494.1972.tb00078.x 4642390

[B89] Miller T. L. Grimes M. G. McMullen J. S. Vogus T. J. (2012). Venturing for others with heart and head: how compassion encourages social entrepreneurship. *Acad. Manag. Rev.* 37 616–640. 10.5465/amr.2010.0456

[B90] Miranda F. Chamorro-Mera A. Rubio S. (2017). Academic entrepreneurship in Spanish universities: an analysis of the determinants of entrepreneurial intention. *Euro. Res. Manag. Bus. Eco.* 23 113–122. 10.1016/j.iedeen.2017.01.001

[B91] Misuraca G. Lipparini F. Kucsera C. (2016). *The role of the Social Economy in promoting Social Investment.* Social Policy Innovation Series. Seville: European Comission, European Union, Available at https://ec.europa.eu/jrc/sites/jrcsh/files/jrc104054.pdf (accessed December 13, 2020).

[B92] Monllor J. (2010). “Social entrepreneurship: a study on the source and discovery of social opportunitie,” in *Values and Opportunities in Social Entrepreneursup*, eds Hockerts K. Mair J. Robinson J. (London: Palgrave Macmillan), 99–120. 10.1057/9780230298026_6

[B93] Morris M. Schindehutte M. (2005). Entrepreneurial Values and the Ethnic Enterprise: An Examination of Six Subcultures. *J. Small Bus. Manag.* 43 453–479. 10.1111/j.1540-62tX.2005.00147.x

[B94] Muñoz P. Kibler E. (2016). Institutional complexity and social entrepreneurship: A fuzzy-set approach. *J. Bus. Res.* 69 1314–1131. 10.1016/j.jbusres.2015.10.098

[B95] Muscat E. J. Whitty M. (2009). Social Entrepreneurship: Values-Based Leadership to Transform Business Education and Society. *Bus. Renaiss. Quar.* 4 31.

[B96] Myers J. Williard K. (2003). Integrating spirituality into counselor preparation: a developmental, wellness approach. *Couns. Val.* 47 142–155. 10.1002/j.2161-007X.2003.tb00231.x

[B97] Nakao K. Nishide Y. (2020). The development of social entrepreneurship education in Japan. *Entrep. Educ.* 3 95–117. 10.1007/s41959-019-00020-5

[B98] Nandram S. (2016). How spirituality, intuition and entrepreneurship go together? *Philo. Manag.* 15 65–82. 10.1007/s40926-016-0029-7

[B99] Nga J. K. Shamuganathan G. (2010). The influence of personality traits and demographic factors on social entrepreneurship start up intentions. *J. Bus. Ethics* 95 259–282. 10.1007/s10551-009-0358-8

[B100] Nicholls A. (2006). *Social Entrepreneurship: New models of sustainable social change.* Oxford: Oxford University Press.

[B101] Nova SBE (2020). *Nova SBE Entrepreneurhsip Hub. A guide to empower all young entrepreneurial minds! Carvavelos.* Nova SBE.

[B102] Obschonka M. Silbereisen R. Cantner U. Goethner M. (2015). Entrepreneurial self-identity: Predictors and effects within the theory of planned behavior framework. *J. Bus. Psyc.* 30 773–794. 10.1007/s10869-014-9385-2

[B103] Oman D. (2015). “Defining Religion and Spirituality,” in *Handbook of the Psychology of Religion and Spirituality*, eds Paloutzian R. Park C. (New York: The Guilford Press), 23–47.

[B104] Ozaralli N. Rivenburgh N. K. (2016). Entrepreneurial intention: antecedents to entrepreneurial behavior in the U.S.A. and Turkey. *J. Glob. Entrep. Res.* 6 1–32. 10.1186/s40497-016-0047-x

[B105] Patzelt H. Shepherd D. A. (2011). Recognizing opportunities for sustainable development. *Entrep. Theory Pract.* 35 631–652. 10.1111/j.1540-6520.2010.00386.x

[B106] Peterman N. E. Kennedy J. (2003). Enterprise Education: Influencing Students’ Perceptions of Entrepreneurship. *Entrep. Theory Pract.* 28 129–144. 10.1046/j.1540-6520.2003.00035.x

[B107] Peterson C. (2000). The future of optimism. *American Psyc.* 55 44–55. 10.1037/0003-066X.55.1.44 11392864

[B108] Pomerantz M. (2003). The business of social entrepreneurship in a “down economy”. *In Bus.* 25 25–30.

[B109] PORDATA (2014). *Dívida e Défice.* Lisboa: Fundação Francisco Manuel dos Santos, Available at https://www.pordata.pt/Portugal/Administrações+Públicas+d%C3%ADvida+bruta+em+percentagem+do+PIB-2786 (accessed December 13, 2020).

[B110] Prieto L. (2010). Influence of proactive personality on social entrepreneurial intentions among African American and Hispanic undergraduate students: The moderating role of hope. *Acad. Entrep. J.* 17 77–96.

[B111] Riaz Q. Farrukh M. Rehman S. Ishaque A. (2017). Religion and Entrepreneurial Intentions: An Empirical Investigation. *Int. J. Adv. App. Sci.* 3 31–36. 10.4324/9781315054292-10

[B112] Roundy P. (2017). Social entrepreneurship and entrepreneurial ecosystems: complementary or disjoint phenomena? *Int. J. Soc. Eco.* 44 1–18. 10.1108/IJSE-02-2016-0045

[B113] Roundy P. Taylor V. Evans W. (2016). Founded by Faith: social entrepreneurship as a bridge between religion and work. *J. Ethics Entrep.* 6 13–27.

[B114] Ruiz-Rosa I. Gutiérrez-Taño D. García-Rodríguez J. (2020). Social entrepreneurial intention and the impact of COVID-19 Pandemic: a structural model. *Sustain.* 12 10.3390/su12176970

[B115] Rust B. Gabriels C. (2011). Spirituality in the workplace: awareness of the human resource function. *Afric. J. Bus. Manag.* 5 1353–1364. 10.5897/AJBM10.1137

[B116] Rutter M. (2012). Resilience as a dynamic concept. *Dev. Psychopathol.* 24 335–344. 10.1017/S0954579412000028 22559117

[B117] Sánchez J. Carballo T. Gutiérrez A. (2011). The entrepreneur from a cognitive approach. *Psicot.* 23 433–438. 21774897

[B118] Sánchez-García J. C. (2010). Evaluación de la Personalidad Emprendedora: validez factorial del Cuestionario de Orientación Emprendedora (COE). *Rev. Latinoamaricana Psico.* 42 41–52.

[B119] Santos S. C. Caetano A. Curral L. (2013). Psychosocial aspects of entrepreneurial potential. *J. Small Bus. Entrep.* 26 661–685. 10.1080/08276331.2014.892313

[B120] Sastre-Castillo M. A. Peris-Ortiz M. Danvila-Del Valle I. (2015). What is different about the profile of the social entrepreneur? *Nonprofit Manag. Leader.* 25 349–369. 10.1002/nml.21138

[B121] Scheier M. F. Carver C. S. (1992). Effects of optimism on psychological and physical well-being: the influence of generalized outcome expectancies. *Health Psyc.* 16 201–228. 10.1007/BF01173489

[B122] Shapero A. Sokol L. (1982). “The social dimensions of entrepreneurship,” in *Encyclopedia of entrepreneurship*, eds Kent C. A. Sexton D. L. Vesper K. H. (Englewood Cliffs, NJ: Prentice-Hall, Inc), 72–90.

[B123] Shinnar R. S. Giacomin O. Jansen F. (2012). Entrepreneurial perceptions and intentions: the role of gender and culture. *Entrep. Theory Pract.* 36 465–493. 10.1111/j.1540-6520.2012.00509.x

[B124] Sieger P. Fueglistaler U. Zellweger T. (2014). “Student entrepreneurship across the globe: a look at intentions and activities,” in *International Report of the Guess – Global University Entrepreneurial Spirit Students’ Survey*, (St Gallen: University of St. Gallen).

[B125] Smith B. Conger M. McMullen J. Neubert M. (2019). Why believe? The promise of research on the role of religion in entrepreneurial action. *J. Bus. Vent. Insights* 11 10.1016/j.jbvi.2019.e00119

[B126] Storey D. (2011). Optimism and chance: the elephants in the entrepreneurship room. *Int. Small Bus. J.* 29 303–321. 10.1177/0266242611403871

[B127] Strack G. Fottler M. (2002). Spirituality and effective leadership in healthcare: Is there a connection? *Front. Health Serv. Manag.* 18:3–17. 10.1097/01974520-200204000-00002 12087690

[B128] Swail J. Down S. Kautonen T. (2014). Examining the effect of ‘entre-tainment’ as a cultural influence on entrepreneurial intentions. *Inter. Small Bus. J. 0* 10.1177/0266242613480193

[B129] Teixeira A. C. (2008). Entrepreneurial potential in chemistry and pharmacy: results from a large sample. *J. Bus. Chem.* 5 48–63.

[B130] Thompson E. R. (2009). Individual entrepreneurial intent: construct clarification and development of an internationally reliable metric. *Entrep. Theory Pract.* 33 669–694. 10.1111/j.1540-6520.2009.00321.x

[B131] Thornton P. H. Ribeiro–Soriano D. Urbano D. (2011). Socio–cultural factors and entrepreneurial activity: An overview. *Int. Small Bus. J.* 29 105–118. 10.1177/0266242610391930

[B132] Tiwari P. Bhat A. K. Tikoria J. (2017). An empirical analysis of the factors affecting social entrepreneurial intentions. *J. Global Entrep. Res.* 7 9. 10.1186/s40497-017-0067-1

[B133] Tracey P. Phillips N. (2007). The Distinctive Challenge of Educating Social Entrepreneurs: A Postscript and Rejoinder to the Special Issue on Entrepreneurship Education. *Acad. Manag. Learn. Educ.* 6 264–271. 10.5465/AMLE.2007.25223465

[B134] Tran A. Von Korflesch H. (2016). A conceptual model of social entrepreneurial intention based on the social cognitive career theory. *Asia Pacific J. Innov. Entrep.* 10 17–38. 10.1108/APJIE-12-2016-007

[B135] Trevelyan R. (2008). Optimism, overconfidence and entrepreneurial activity. *Manag. Dec.* 46 986. 10.1108/00251740810890177

[B136] Urban B. (2010). Cognitions and motivations for new venture creation decisions: linking expert scripts to self-efficacy, a South African study. *Int. J. Hum. Res. Manag.* 21 1512–1530. 10.1080/09585192.2010.488457

[B137] Vamvaka V. Stoforos C. Palaskas T. Botsaris C. (2020). Attitude toward entrepreneurship, perceived behavioral control, and entrepreneurial intention: dimensionality, structural relationships, and gender differences. *J. Innov. Entrep.* 9 1–26. 10.1186/s13731-020-0112-0

[B138] Vasakarla V. (2008). A study on social entrepreneurship and the characteristics of social entrepreneurs. *ICFAI J. Manag. Res.* 7 32–40.

[B139] Waddock S. Steckler E. (2012). “Wisdom, spirituality, social entrepreneurs, and self-sustaining practices: what can we learn from difference makers?,” in *Handbook of faith and spirituality in the workplace*, ed. Neal J. (New York: Springer), 285–301. 10.1007/978-1-4614-5233-1_18

[B140] Ward A. Hernández-Sánchez B. Sánchez-García J. C. (2019). Entrepreneurial potential and gender effects: the role of personality traits in university students’ entrepreneurial intentions. *Front. Psyc.* 10:2700. 10.3389/fpsyg.2019.02700 31866902 PMC6905410

[B141] Wood R. Bandura A. (1989). Social cognitive theory of organizational management. *Acad. Manag. Rev.* 14 361–384. 10.5465/AMR.1989.4279067

[B142] Yang R. Meyskens M. Zheng C. Hu L. (2015). Social entrepreneurial intentions: China versus the USA – Is there a difference? *Inter. J. Entrep. Innov.* 16 253–267. 10.5367/ijei.2015.0199

[B143] Yap S. Othman M. Wee Y. (2013). The fallacy of one-dimensional theory of planned behavior structure in predicting health behavior. *Int. J. Behav. Health. Res.* 4 26–44. 10.1504/ijbhr.2013.054516

[B144] Yitshaki R. Kropp F. (2016). Motivations and Opportunity recognition of social entrepreneurs. *J. Small Bus. Manag.* 54 546–565. 10.1111/jsbm.12157

[B145] Zhao H. Seibert S. E. Lumpkin G. T. (2010). The relationship of personality to entrepreneurial intentions and performance: a meta-analytic review. *J. Manag.* 36 381–404. 10.1177/0149206309335187

[B146] Zinnbauer B. J. Pargament K. I. (2005). “Religiousness and spirituality,” in *Handbook of the psychology of religion andspirituality*, eds Paloutzian R. F. Park C. L. (New York: The Guilford Press), 21–42. 10.4324/9780203821572-5

[B147] Zsolnai L. (2014). Emprendedorismo guiado por la espiritualidade. *Rev. Cul. Eco.* 32 35–46.

[B148] Zsolnai L. Illes K. (2017). Spiritually-inspired creativity in business. *Int. J. Soc. Eco.* 44 1–20. 10.1108/ijse-06-2015-0172

